# When the Sailor’s Handshake Fails: A Case of Syphilitic Hepatitis in an HIV Patient With Nonspecific Liver Biopsy and Atypical Cutaneous Presentation

**DOI:** 10.7759/cureus.22802

**Published:** 2022-03-03

**Authors:** Rachel Dawson, Davena Zhang, Navid Salahi, Daniel Kashani

**Affiliations:** 1 Medical School, St. George’s University, Saint George, GRD; 2 Internal Medicine, State University of New York Downstate Medical Center, New York, USA; 3 Pathology, State University of New York Downstate Medical Center, New York, USA; 4 Hospital Internal Medicine, Mayo Clinic, Rochester, USA

**Keywords:** biopsy, rash, hiv, hepatitis, syphilis

## Abstract

Secondary syphilis has variable systemic manifestations, impersonating the presentation of more common pathologies, deceiving clinicians, and creating a difficult-to-diagnose patient. The case discussed combines hepatic syphilis with an uncommon syphilitic dermatologic presentation in a patient with HIV and a history of hepatitis A and B. Due to the challenge of diagnosis, the relative ease of confirming the diagnosis with serological assays, and reversibility of hepatic injury, the inclusion of syphilitic hepatitis on a differential diagnosis of hepatitis is warranted.

## Introduction

Syphilitic hepatitis is an uncommon, potentially underreported manifestation of early and secondary syphilis. Spirochete dissemination has variable systemic manifestations, including hepatic, gastrocolic, renal, and dermatologic injury [1]. Inflammatory response to spirochetes may be responsible for pathology, as evidenced by lesion histopathology displaying various inflammatory infiltrates [2]. The degree of injury and clinical presentation may be influenced by individual immune-responsiveness, specifically augmented responses in HIV patients [3]. Hepatic pathology is more easily observed in congenital syphilis than acquired syphilis [4]. Acquired syphilis typically displays the characteristic non-pruritic maculopapular rash accompanied by signs of hepatic injury on liver function tests, most notable elevations of alkaline phosphatase (ref: 35-145 U/L). This case study combines an uncommon presentation of intensely pruritic and atypical dermatologic symptoms, comorbid HIV infection, and a liver biopsy negative for spirochetes.

## Case presentation

The patient is a 48-year-old male with a past medical history of HIV on elvitegravir, cobicistat, emtricitabine, and tenofovir combination pill (GENVOYA) (inconsistently adherent), polysubstance use disorder (crystal methamphetamine, marijuana, tobacco), ureteral stent placement for recurrent kidney stones and glaucoma who presented to the emergency department (ED) with a one-month history of recurrent skin abscesses and pruritis most significant on the right shin. He also complained of generalized weakness and decreased oral intake. One month prior to presentation, the patient's CD4 count was 398 with an undetectable HIV viral load.

He was afebrile but mildly tachycardic to 110 in the ED. On admission his alanine aminotransferase (ALT) was 137 U/L (0-41 U/L), aspartate aminotransferase (AST) was 83 U/L (0-50 U/L), alkaline phosphatase (ALP) was 976 U/L (35-145 U/L) and total bilirubin 1.5 mg/dL (0-1.2 mg/dL). Multiple services, including infectious disease, dermatology, and hepatobiliary, were consulted to evaluate the initial findings.

**Figure 1:**
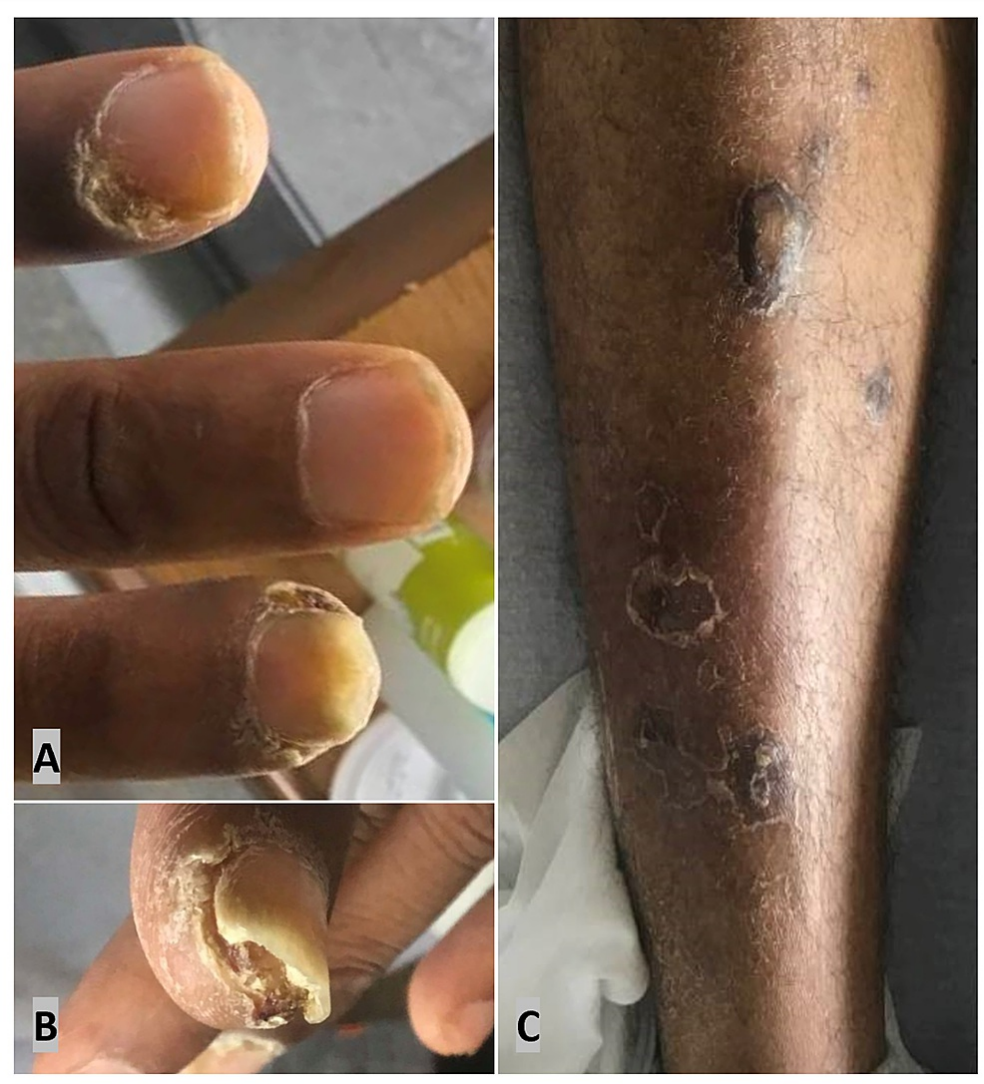
Skin lesions on the fingernails and lower extremity (A) Left second (top) and fourth (bottom) fingernails. Ulceration with crusting is suggestive of ruptured paronychia or onychomycosis. Yellow subungual debris and separation of the nail bed on the second finger. (B) Left second finger shows the separation of the nail bed. (C) Right lower extremity lesions. Scattered, skin-colored, and hyperpigmented papules with a background of pitted scarring and evident excoriations.

Dermatologic exam of the distal left second and fourth digit found painful ulcerations without purulent discharge and separation of the nail bed on the second digit (Figures 1A, 1B). Multiple healing lesions and raised, violaceous nodules with sporadic lichenification were present on bilateral lower extremities (Figure 1C). Similar lesions were discovered on the scalp, trunk, and upper extremities. Notably, the palms and soles were free from cutaneous pathology. The perianal region displayed few pedunculated papules. Right anterior tibial cellulitis with a small abscess (+methicillin-resistant Staphylococcus aureus) was drained at the bedside and treated with trimethoprim/sulfamethoxazole. No Osler nodes, splinter hemorrhages, or jaundice were visualized. 

**Figure 2:**
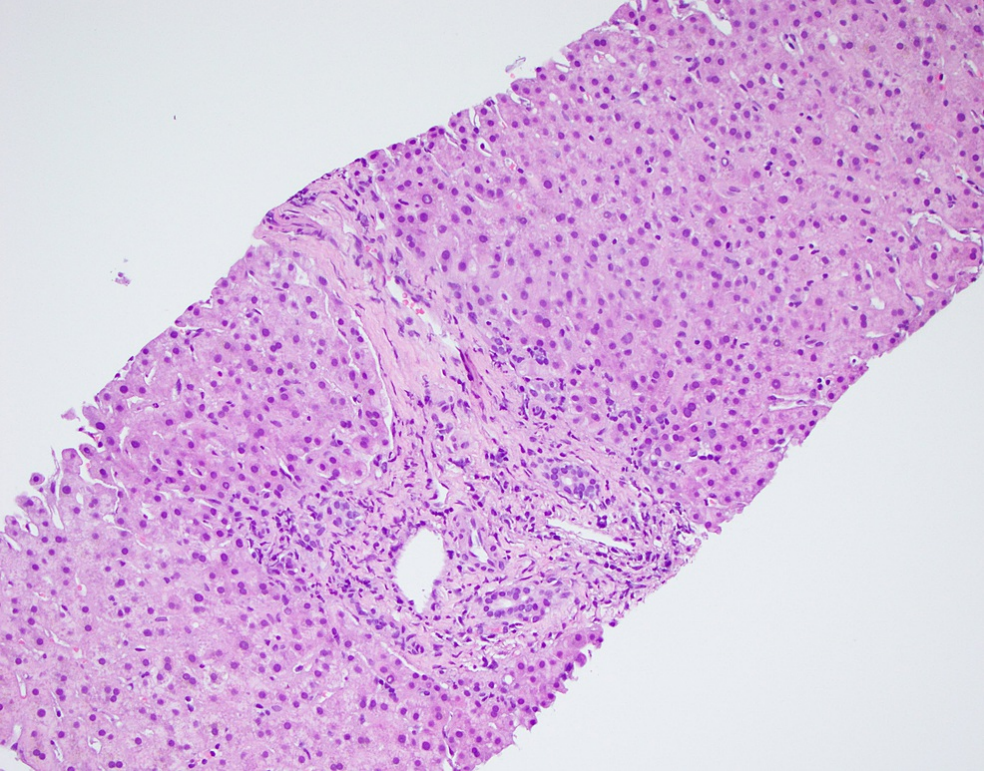
Liver core biopsy displaying predominantly neutrophilic mixed inflammation of the portal and periportal hepatocytes with patchy hepatocellular necrosis. Negative for granulomas and immuno-histochemical markers negative for spirochetes. (H&E, 20×) H&E: Hematoxylin and Eosin

**Figure 3:**
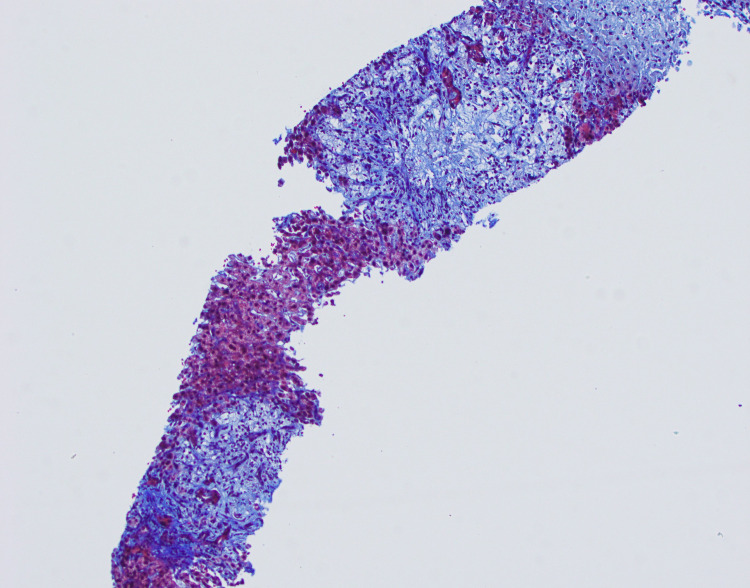
Trichrome stain of liver biopsy, significant for fibrosis.

Liver function tests (LFT) continued to rise during admission without an obvious etiology. Zenith values for liver enzymes were ALP 1,143 U/L, total bilirubin 4.1 mg/dL, AST 158 U/L, and ALT 228 U/L. The patient did not have any abdominal complaints. Imaging of the abdomen by CT scan failed to delineate an etiology, prompting a liver biopsy (Figures 2, 3).

**Figure 4:**
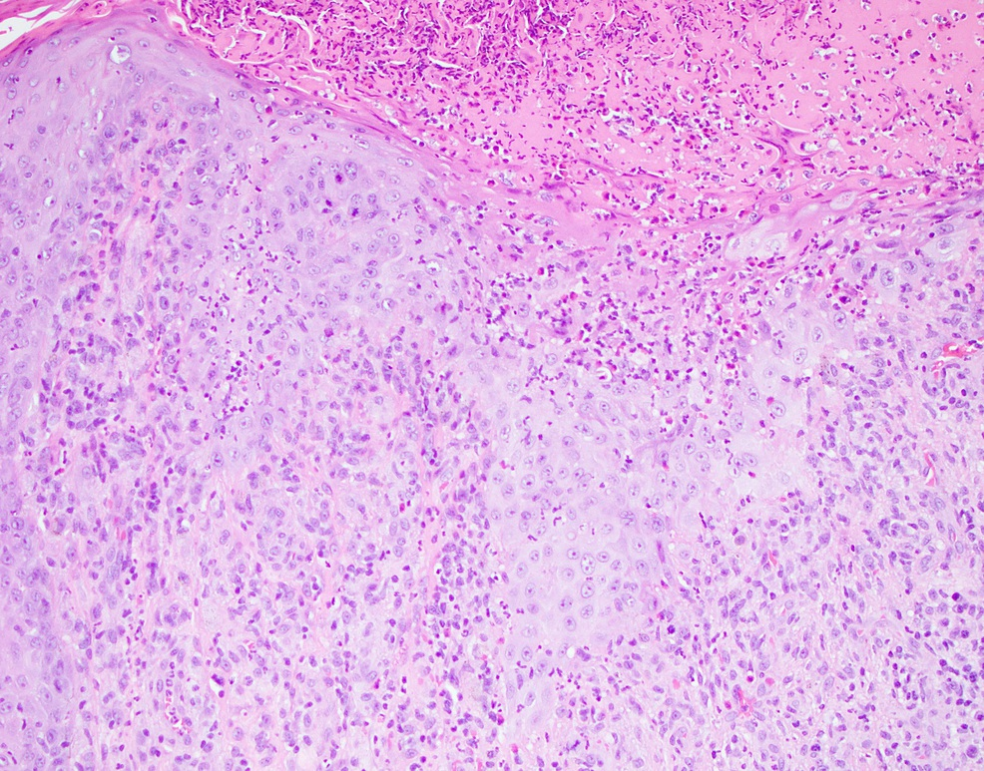
Skin shave biopsy from vertex scalp shows irregular psoriasiform hyperplasia, abundant neutrophils in stratum corneum and epidermis, and dense lymphoplasmacytic infiltration that obscures dermo-epidermal junction (H&E, 20×). H&E: Hematoxylin and Eosin

**Figure 5:**
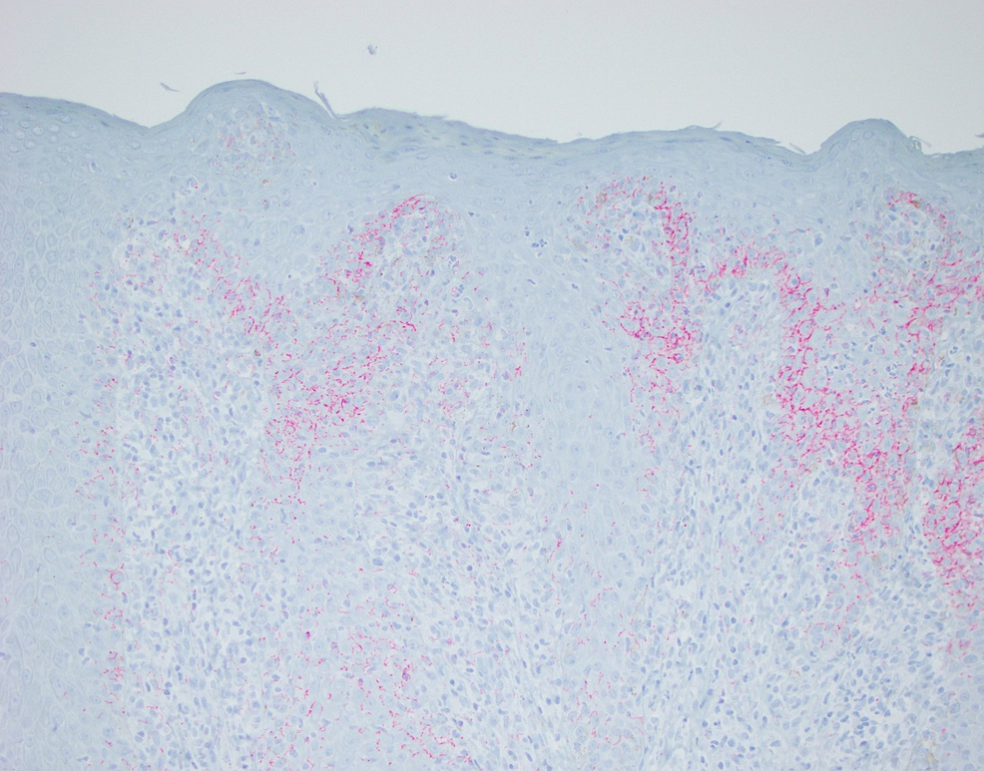
Skin shave biopsy stained with red chromogen, highlighting Treponema pallidum at the dermo-epidermal junction.

With acute biliary pathology ruled out, less common causes of hepatitis were considered. Laboratory investigations found: rapid plasma reagin (RPR) reactive with a titer of 1:512; hepatitis A IgG antibody reactive; hepatitis B core and surface antigen-positive; anti-smooth muscle antibody IgG elevated 1:40 (normal <1:20), and anti-nuclear antibody (ANA) elevated (1:320) and speckled (<1:80). Other autoimmune investigations were negative including, anti-DNA, Smith, mitochondrial, and cryoglobulin. Skin shave biopsies of ulcerations found psoriasiform hyperplasia, numerous neutrophils, and stained positive for spirochetes (Figures 4, 5).

Following treatment with penicillin G benzathine 2.4 million units, LFTs began to downtrend. During hospitalization, the patient received two doses with the following final labs: AST 48 U/L (0-50 U/L), ALT 102 U/L (0-41 U/L), ALP 731 U/L (35-145 U/L), and total bilirubin 1.1 mg/dL (0-1.2 mg/dL). There is no record of the patient receiving the third dose, and they were lost to follow up.

## Discussion

While hepatitis remains an uncommon manifestation of syphilis, hepatic involvement has been a known sequela since 1585, as described by Paracelsus [3]. Kellock and Laird noted in 1956 that the diagnosis was predominantly clinical and supported by positive serological evidence, response to treatment, and lack of evidence for alternate etiologies [5]. These early observations are reflected in the criteria outlined by Mullick et al. in 2004 [3]: (1) abnormal liver function tests (LFTs); (2) positive syphilis serologies (RPR and fluorescent treponemal antibody absorption [FTA-Abs] or microhemagglutination assay for Treponema pallidum antibodies [MHA-TP]); (3) evidence to rule out other etiologies of hepatic injury and (4) clinical improvement of LFTs following initiation of pharmacologic therapy. Liver function tests often present a cholestatic pattern more often than hepatocellular, notably with significant elevation in ALP [2, 3, 6]. However, patients can present with variable liver function tests and physical presentations, further challenging diagnosis and requiring a high index of suspicion (Table 1). A thorough social history can be paramount in identifying risk factors for acquired syphilis in patients without a history of infection. Risk factors noted by the United States enters for Disease Control and Prevention (CDC) include sexual activity with multiple or anonymous partners, men who have sex with men, and individuals living with HIV. A higher incidence of syphilitic hepatitis in patients with HIV has been noted in the literature [6].

**Table 1:** Comparison of patients described in referenced case reports Most case reports reviewed described rashes typical of syphilis and laboratory findings suggestive of syphilitic hepatitis, specifically marked elevation in ALP. Liver biopsy findings were variable when performed. Pruritic rashes were described by Baveja et al. [7] and Cordoso et al. [10]. Abbreviations: F = female, M = male, HIV = Human Immunodeficiency Virus, HAART= Highly active antiretroviral therapy, AST = aspartate aminotransferase, ALT = alanine transaminase, ALP = alkaline phosphatase, GGT = gamma glutamyl aminotransaminase, TBil = total bilirubin, RPR = rapid plasma reagin, VDRL = venereal disease research laboratory test, FTA-Abs = fluorescent treponemal antibody absorption IgM, LFT = Liver function test, TPPA= Treponema pallidum particle assay, TPHA = treponema pallidum hemagglutination assay, AMA = anti-mitochondrial antibody, ASMA = anti-smooth muscle antibody.

Case Reports	Age, Sex	Past Medical History	Initial presentation	Physical Exam	Lab findings	Liver biopsy findings	Response to therapy
Shinn et al. [1]	44, F	No significant medical history	Fever, sore throat, myalgia, abdominal pain, nausea, early satiety	Diffuse pruritic rash	AST 61 IU/L ALT 173 IU/L ALP 284 IU/L TBil 1.2 mg/dL RPR 1:128 AMA 57.3 U/L ASMA 36 U/L IgG 2,005 mg/dL	Cholestatic hepatitis, prominent bile duct injury, inflammation, stage 1/4 fibrosis No spirochetes	Doxycycline Asymptomatic Normalization of liver enzymes, ASMA & total IgG
Marcos et al. [2]	48, M	History of unprotected heterosexual intercourse 2 months prior to presentation.	2- week epigastric tenderness, asthenia	Erythematous, maculopapular rash	AST 154 IU/L ALT 324 IU/L ALP 390 IU/L GGT 1384 IU/L VDRL 1:64 FTA-Abs positive	Not reported	Penicillin G Asymptomatic Normalization of liver enzymes Nonreactive VDRL
Mullick et al. [3]	39, M	15-year history of HIV (CD4 455/mm^3^) infection	3- week dull intermittent right upper quadrant pain.	Scleral icterus. Generalized maculopapular rash. Mild RUQ tenderness	ALP 727 IU/L TBil 4.1 mg/dL RPR 1:4096 FTA-Abs positive Urine 2+ bilirubin	Not done	Penicillin G Asymptomatic Normalization of liver enzymes Nonreactive RPR
Huang et al. [4]	47, M	None reported	Jaundice, nonpruritic rash all over the body	Skin and scleral icterus	AST 161 IU/L ALT 359 IU/L ALP 580 IU/L TBil 75.1 umol/L GGT 883 IU/L RPR 1:32 TPPA 1:38	Granulomatous hepatitis, stage 2 inflammation, stage 1 fibrosis, mild hepatic lobule inflammation. Micro-granulomas with coagulation necrosis in the portal area	Penicillin G Asymptomatic Normalization of liver enzymes after 2 months
Pizzarossa, Rebella [6]	Unknown age, F	Partially controlled asthma, impaired fasting glycemia	Fever, myalgia, headache, generalized nonpruritic rash	Macular, erythematous rash Hepatosplenomegaly	AST 321 IU/L ALT 247 IU/L ALP 721 IU/L TBil 1.07 mg/dL GGT 550 IU/L VDRL 32 IU TPHA positive	Not done	Penicillin G Asymptomatic Normalization of liver enzymes after 1 week, VDRL 8 IU after 2 weeks
Baveja et al. [7]	39, M	3- year alcohol use disorder	10 days: pain in upper abdomen, low-grade fever, anorexia, malaise, dark-colored urine	Jaundice, non-tender hepatomegaly Diffuse, pruritic, erythematous papules & plaques Positive Buschke-Olendroff sign	AST 175 IU/ml ALT 357 IU/ml ALP 536 IU/ml TBil 3.5 mg/dL VDRL 1:16 TPHA positive	Kupffer cell hyperplasia, lymphocytic & neutrophilic infiltration of portal tracts with mild neutrophilic infiltrate in hepatic lobules	Penicillin G Asymptomatic Normalization of liver enzymes
Makker et al. [8]	51, M	Diabetes mellitus, osteoarthritis, use of phencyclidine and cannabinoids	Abdominal pain, bright red blood per rectum, non-bilious vomiting	Epigastric and peri-umbilical pain Copper colored papules & macules Mild bilateral pitting pedal edema	AST 50 IU/L ALT 91 IU/L ALP 274 IU/L TBili 0.3 mg/dL RPR 1:1,024	Chronic hepatitis with mild activity, stage 1 peri-portal fibrosis	Penicillin G Not reported
Kern et al. [9]	54, M	HIV infection (CD4 336/mm^3^), hypertension, hyperlipidemia, diabetes mellitus	Diffuse abdominal pain and vomiting	Jaundice, scleral icterus Diffuse nonpruritic erythematous, macular rash on torso, back extremities, palms	AST 91 IU/L ALT 120 IU/L ALP 832 IU/L TBil 6.4 mg/dL RPR 1:256 Positive Anti-M2 AMA IgG 83.5 U	Not done	Doxycycline Asymptomatic Normalization of LFTs after 2 months Negative RPR AMA IgG 16.4
Cardoso et al. [10]	46, M	5-year history of HIV (CD4 516/mm^3^) on HAART, unprotected sexual intercourse	Not reported	Pruritic maculopapular rash in the dorsal region and proximal region of lower limbs	ALP 308 U/L RPR 512 FTA-Abs positive	Mononuclear inflammatory infiltrate of portal spaces Discrete lobular necro-inflammatory activity with dispersed acidophilic bodies & focal epithelioid granulomatous aggregates without necrosis	Penicillin G Asymptomatic Normalization of LFTs

Hepatic dysfunction in the context of HIV may be related to increased rates of coinfection with hepatotropic pathogens, the pathophysiology of which may be related to immune deficiency permissive of pathogenic dissemination [3]. While the patient discussed was reactive for both hepatitis A and B during admission, his presentation suggested an alternate etiology for serologic findings based on medical history. Hepatitis reactivity was discovered on prior outpatient workups without concomitant elevations in LFTs. Further, liver enzymes were not elevated while adherent to GENVOYA, an antiretroviral with known hepatotoxicity. As previously reported [1], due to the cross-reactivity of specific antibodies with the *T. pallidum* membrane, some diagnostic workup, including anti-smooth muscle antibodies, may be falsely elevated and will return to normal upon syphilis-targeted therapy. Our patient met previously outlined criteria by combining serological evidence of hepatic injury, infection with syphilis, exclusion of alternate etiologies, and response to treatment. A biopsy is not required to confirm the diagnosis of syphilitic hepatitis, especially with a positive response to treatment, because cellular changes are variable, and spirochetes may not be visible [2, 3, 6]. Liver biopsy for our patient was suggestive of mixed inflammation with patchy necrosis and negative for granulomas and spirochetes (Figure 2). Other cases reported granulomas and inflammatory cells in portal tracts and hepatic lobules [3-8]. Our case presented a patient with primarily dermatologic complaints whose evaluation led to the diagnosis of syphilitic hepatitis. Rashes and dermatologic lesions are more often reported as painless and non-pruritic [1-4, 8, 9]. Sparse cases reported pruritic rashes but not subjective pain [4, 10]. Interestingly, immunohistochemical staining of skin biopsies highlighted numerous spirochetes, further supporting the diagnosis of secondary syphilis (Figure 5). 

Despite the challenge of diagnosis, the relative ease of confirming the diagnosis with serological assays and reversibility of hepatic injury warrant inclusion on a differential diagnosis of hepatitis. With rates of syphilis on the rise, suspicion should be increased in patients with a history of risk factors for syphilis and in communities with high rates of transmission.

## Conclusions

Clinicians should highly suspect syphilitic hepatitis in patients with risk factors (HIV, men having sex with men, multiple anonymous sexual partners) and elevated LFTs (predominantly the elevated ALP) when alternative etiology is unlikely. The initial syphilitic hepatitis presentation may be symptomatic or asymptomatic and with or without concurrent skin lesions. The clinician should always carefully examine the patient's skin and assess the need for a biopsy. Having syphilitic hepatitis as part of differential and obtaining syphilis serology as part of the initial workup may expedite the diagnosis and treatment plan for the patient in this clinical context. Normalizing LFTs after syphilis-targeted treatment may reasonably spare liver biopsy and its complications.
